# Replication and Active Demethylation Represent Partially Overlapping Mechanisms for Erasure of H3K4me3 in Budding Yeast

**DOI:** 10.1371/journal.pgen.1000837

**Published:** 2010-02-05

**Authors:** Marta Radman-Livaja, Chih Long Liu, Nir Friedman, Stuart L. Schreiber, Oliver J. Rando

**Affiliations:** 1 Department of Biochemistry and Molecular Pharmacology, University of Massachusetts Medical School, Worcester, Massachusetts, United States of America; 2 School of Computer Science and Engineering, The Hebrew University, Jerusalem, Israel; 3 The Alexander Silberman Institute of Life Sciences, The Hebrew University, Jerusalem, Israel; 4 Broad Institute of Harvard and Massachusetts Institute of Technology, Cambridge, Massachusetts, United States of America; The University of North Carolina at Chapel Hill, United States of America

## Abstract

Histone modifications affect DNA–templated processes ranging from transcription to genomic replication. In this study, we examine the cell cycle dynamics of the trimethylated form of histone H3 lysine 4 (H3K4me3), a mark of active chromatin that is viewed as “long-lived” and that is involved in memory during cell state inheritance in metazoans. We synchronized yeast using two different protocols, then followed H3K4me3 patterns as yeast passed through subsequent cell cycles. While most H3K4me3 patterns were conserved from one generation to the next, we found that methylation patterns induced by alpha factor or high temperature were erased within one cell cycle, during S phase. Early-replicating regions were erased before late-replicating regions, implicating replication in H3K4me3 loss. However, nearly complete H3K4me3 erasure occurred at the majority of loci even when replication was prevented, suggesting that most erasure results from an active process. Indeed, deletion of the demethylase Jhd2 slowed erasure at most loci. Together, these results indicate overlapping roles for passive dilution and active enzymatic demethylation in erasing ancestral histone methylation states in yeast.

## Author Summary

Organisms can inherit information beyond DNA sequence, a phenomenon known as epigenetic inheritance. It is widely believed that chromatin marks provide a carrier for epigenetic information, a hypothesis that is less-supported than generally believed. In this study, we measure the erasure of a “memory” mark of active transcription, H3K4me3. We find that this signal-responsive chromatin mark largely returns to baseline levels within one generation. Furthermore, we find that this erasure occurs during S phase in a manner consistent with its loss during replication, yet we find that replication only contributes modestly to the erasure process. Instead, active enzymatic demethylation is required for erasure. Together, these results show that even chromatin states widely associated with epigenetic memory are only maintained in the ongoing presence of activating signals, and are not generally heritable.

## Introduction

The histone proteins that package eukaryotic genomes into chromatin are subject to a huge number of covalent modifications [1]–[4]. Histone modifications have wide-ranging effects on cellular physiology, and play roles in gene expression, RNA transcript structure, meiotic recombination, and many other processes.

Histone H3 lysine 4 trimethylation (H3K4me3) is an active chromatin mark found at the 5′ ends of coding regions at levels that scale with transcription rate [5]–[9]. Unlike histone acetylation, which has a typical half-life on the order of minutes [10],[11], histone methylation is considered more stable: in yeast, H3K4me3 is rapidly deposited by RNA polymerase-associated Set1 when genes are activated, but after gene repression the mark persists well after Set1 has dissociated from the gene ([1], see below) leading to the suggestion that it forms a “memory mark” of recent transcription. Indeed, H3K4 methylation is associated with the Trithorax complex of genes involved in cell state memory in *Drosophila* [2], indicating that in this context it plays a role in epigenetic inheritance.

Several single-locus studies in yeast indicate that H3K4 methylation is lost upon gene repression. In one study, H3K4 methylation over the *GAL1-10* locus decreased ∼2-fold by one hour after glucose repression, but did not return to baseline levels until 5 hours of repression [1]. More recently, the partial loss of methylation observed 75 minutes after dextrose addition was shown to be dramatically slowed, but not eliminated, in the absence of the histone demethylase *JHD2/KDM5* [12]. At the epigenetically-regulated heterochromatic loci in budding yeast [13],[14], high levels of H3K4me3 are observed in mutants lacking the Sir silencing complex. Interestingly, even at these loci, H3K4me3 is erased over several generations (∼7.5 hours) upon reintroduction of a functional Sir complex [15]. This loss of H3K4me3 was suggested to rely predominantly on enzymatic demethylation, although a modest effect of replication in H3K4me3 erasure was not ruled out [15]. Together, these results indicate that H3K4 methylation is not maintained in budding yeast after removal of an activating stimulus, even at loci known to be subject to epigenetic regulation.

However, these conclusions come from a small number of unusual genomic loci (largely the *GAL* genes and heterochromatic loci), and their generality is unknown. Furthermore, in different studies H3K4me3 erasure has been suggested to occur predominantly via passive replication-mediated dilution [1], enzymatic demethylation [12], or both [15], raising the mechanistic question of how H3K4me3 erasure occurs. In other words, how long does elevated H3K4me3 at an active gene last after gene repression, does it vary between genomic loci, and how is memory lost or erased? Here, we investigate temporal aspects of H3K4me3 memory of recent transcription during the cell cycle in budding yeast. We find that H3K4 methylation induced by three different stimuli (alpha factor, high temperature, and galactose) is rapidly erased after removal of the activating stimulus. We find that this erasure typically occurs during S phase in a manner consistent with replication, but we find that replication only contributes modestly to the bulk of H3K4me3 erasure. Instead, the demethylase Jhd2/Kdm5 is responsible for most H3K4 erasure.

## Results

### Periodic H3K4 Methylation during the Cell Cycle

How long might H3K4 methylation persist after gene repression, and how does it differ at different genes? We carried out genome-scale profiling of H3K4me3 patterns during cell cycle progression using a 20 bp resolution tiling microarray covering 4% of the yeast genome [6],[16]. We synchronized yeast using one of two methods (Table S1 and Table S2). In one, yeast carrying the temperature-sensitive *cdc28-13* allele were arrested at 37 degrees, then rapidly returned to 25 degrees to allow re-entry into the cell cycle. Cells were fixed every 10 minutes for ∼1.5 cell cycles, and H3K4me3 levels and nucleosome occupancy were measured. The alternative synchrony protocol involved alpha factor arrest of *bar1Δ* yeast, followed by release into fresh media lacking alpha factor, with fixation every 5 (early) to 10 (late) minutes for ∼3 cell cycles. Gene expression analysis (Figure S1) showed good synchrony in both cell cycles, with a notable distinction being the more rapid cycling of cells released from alpha factor block (CCA). Furthermore, we recently reported mapping of H3K56ac during the *cdc28-13* “temperature shift” cell cycle (CCTS), with S phase peaks of H3K56ac demonstrating good synchrony [17].

**Figure 1:**
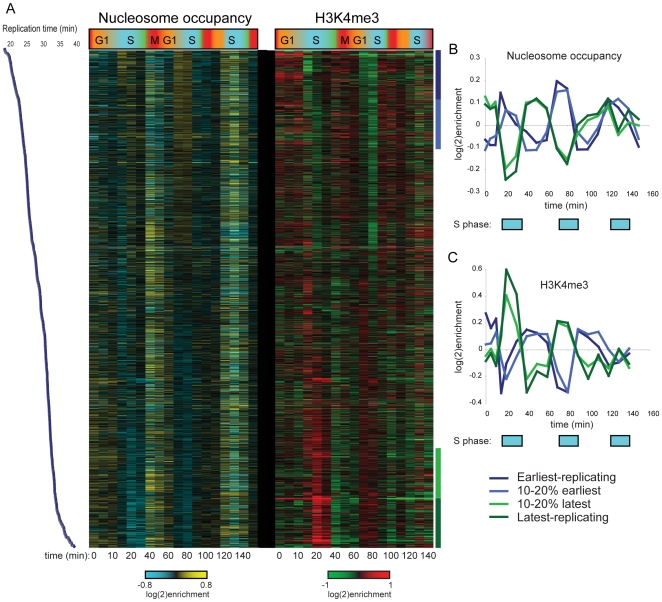
Cell cycle variation in H3K4me3 patterns. (A) Nucleosome occupancy (left) and H3K4me3 levels (right) were measured in yeast undergoing synchronous release from alpha factor arrest. Each row represents one of 2288 nucleosomes, and nucleosomes are ordered by previously measured replication time [18]. Note that replication timing and our cell cycle are different experiments and thus the two time scales should not be considered identical. (B) Average nucleosome occupancy for four 10% bins of nucleosomes over the course of CCA. Early-replicating nucleosomes exhibit increased occupancy during S phase, while late-replicating nucleosomes show the converse pattern. (C) Average H3K4me3 levels as in (B). K4 methylation levels show an inverse pattern to occupancy profiles, consistent with newly-incorporated nucleosomes lacking H3K4me3.

We initially analyzed H3K4me3 patterns to search for correlations with cell cycle-regulated gene expression. As predicted (due to the long life of H3K4me3), we found no correlation between a gene's peak phase of expression and variation in H3K4me3 levels of nearby nucleosomes. Most genes exhibiting maximal expression during G1, for example, did not exhibit periodic 5′ H3K4me3 over the time courses (not shown). However, we did observe periodic variation in nucleosome occupancy and H3K4me3 levels at a number of nucleosomes, and found that this variation was correlated with replication timing [18] of the region in question (Figure 1A shows data for CCA). Specifically, nucleosome occupancy varied markedly through the cell cycle in early and late-replicating regions of the genome, with increased occupancy of early-replicating regions during S phase (Figure 1B). H3K4me3 levels at the same sets of nucleosomes showed cell cycle variation that was precisely out of phase with nucleosome occupancy (Figure 1C). Similar results, of lesser magnitude, were observed in the CCTS dataset (Figure S2). The variation in nucleosome occupancy can be ascribed to partial genomic replication at intermediate time points – early in S phase, early-replicating regions of the genome will be present in two copies per cell, and since nucleosome assembly after replication is rapid [19], this will result in increased nucleosome occupancy of these regions relative to late-replicating domains. The variation in H3K4me3 is consistent with previous reports that newly-incorporated nucleosomes lack H3K4me3 [20], resulting in relative enrichment of late-replicating nucleosomes with H3K4me3 when early-replicating regions have been half-assembled into H3K4me3-lacking nucleosomes. In other words, our results are consistent with an intermediate chromatin assembly state at early-replicating regions where the genome has been duplicated and assembled into undermethylated chromatin, before methylation is restored during the next round of transcription.

### H3K4 Methylation Induced during Synchronization Is Erased during S Phase

**Figure 2:**
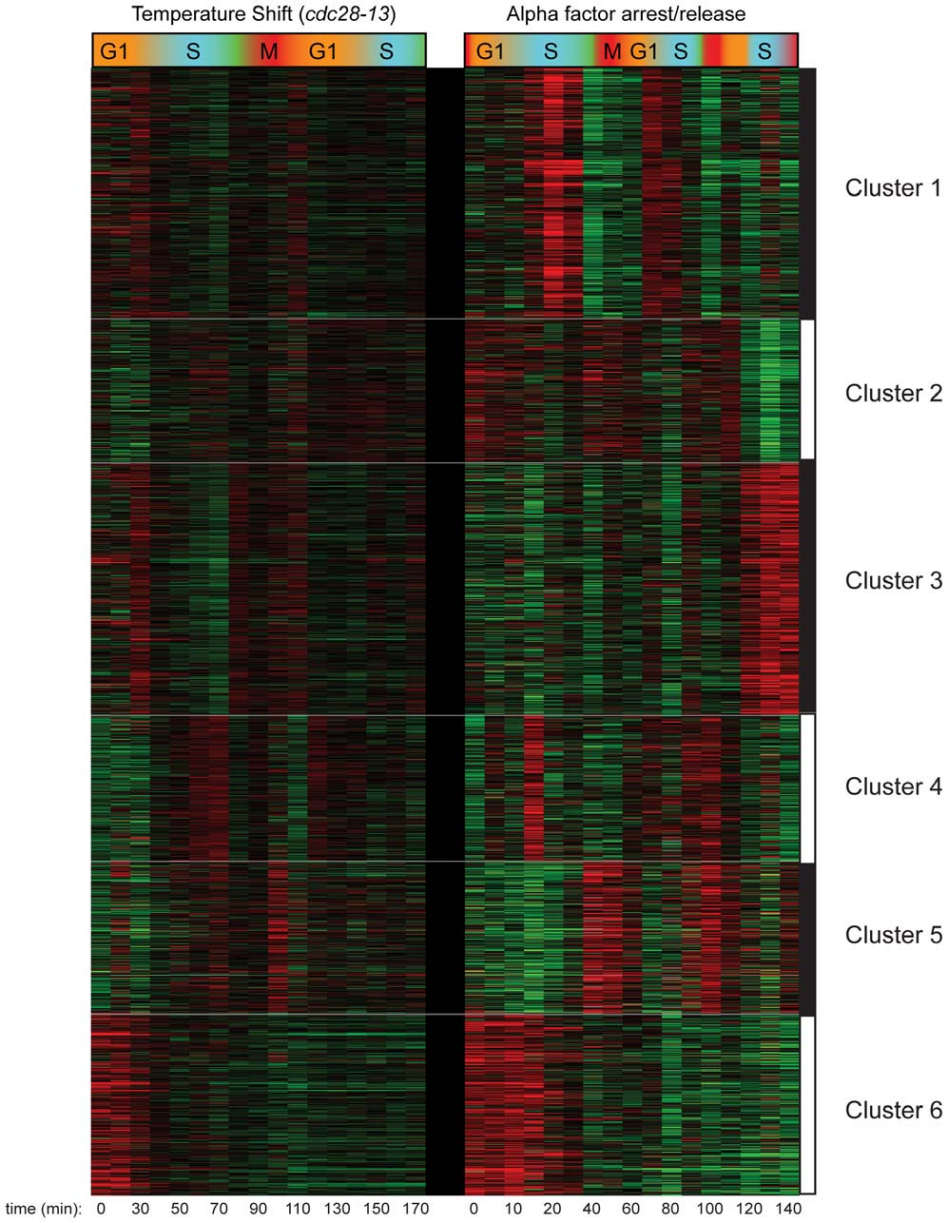
Clustering of CCTS and CCA H3K4me3 patterns. Microarray measurements of H3K4me3 for each nucleosome was centered to zero for each time course to emphasize relative variation in methylation levels, and concatenated data for both time courses was subject to k-means clustering with k = 6.

**Figure 3:**
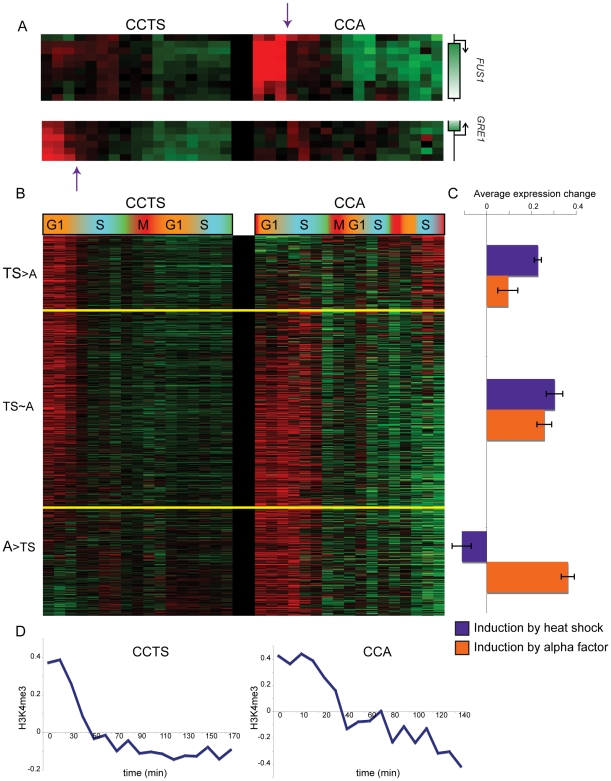
Nucleosomes methylated during cell cycle arrest lose H3K4me3 during the first S phase after release. (A) Examples of genomic loci that have high early H3K4me3 that is largely lost during S phase in only one of the two cell cycles. *FUS1* is an alpha-inducible gene, *GRE1* is a stress response gene. (B) Nucleosomes that match Cluster 6 (r≥0.5) from Figure 2 in either of the two cell cycles (858 total), sorted according to the cell cycle where they best match the Cluster 6 profile. (C) Average heat shock induction levels (blue) or alpha factor induction levels (orange) for the three bins of nucleosomes as shown in (B). (D) Average H3K4me3 patterns over the cell cycle for nucleosomes matching Cluster 6 for CCTS (left) and CCA (right).

Further analysis of the combined cell cycle methylation data for both CCA and CCTS by k-means clustering revealed an interesting, nonperiodic cluster of hundreds of nucleosomes exhibiting high levels of H3K4me3 from arrest until the first S phase, when H3K4me3 levels dropped precipitously (Figure 2, Cluster 6). Genes associated with these “Cluster 6” nucleosomes in CCTS were not enriched for periodic expression during the cell cycle, but we noticed that many of the genes were previously shown to be induced during heat stress [21] (Figure 3A). We reasoned that since these genes would be expressed at relatively high levels during the arrest at 37 degrees but would return to lower levels at 25 degrees, the pattern observed might indicate the process of disassembly of the active chromatin state induced by the cell cycle arrest protocol (Figure S3). Since different methods of cell cycle arrest induce different gene expression patterns, this hypothesis could be tested by examining cell cycle synchrony by a different arrest/release method such as alpha factor synchronization. While many nucleosomes showed similar behavior in both time courses (Figure 2, Cluster 6), a notable difference can be seen at the pheromone response gene *FUS1* (Figure 3A). *FUS1* is strongly induced by alpha factor treatment [22] and exhibits high levels of H3K4me3 during alpha factor arrest, but its K4 methylation levels drop dramatically during early S phase (arrow). This does not occur in the CCTS time course, consistent with the idea that the S phase drop-off represents disassembly of the activated chromatin state. This active state erasure is not specific to H3K4me3 – we have also mapped “active” acetylation marks H3K14ac and H2AK7ac in a separate CCTS, and they are erased at Cluster 6 nucleosomes within 10 minutes, as expected (not shown) [10].

We explored H3K4me3 erasure more broadly by identifying nucleosomes that exhibited S phase H3K4me3 loss in at least one of the two time courses (Materials and Methods), then divided this set of nucleosomes into three groups: TS>A for nucleosomes with greater S phase H3K4me3 loss in CCTS than in CCA, A>TS for the converse, and TS∼A for nucleosomes with similar S phase loss in both time courses (Figure 3B). As predicted, TS>A nucleosomes were associated with more heat-inducible genes than A>TS nucleosomes, which were associated with more alpha factor-responsive genes (Figure 3C). It is important to note that only a subset of genes exhibiting this S phase drop are induced by the relevant stimulus – in some cases, the nucleosome in question is H3K4 methylated despite being associated with a gene whose RNA level is not increased by the arrest. We do not fully understand the role of K4 methylation at these nucleosomes, although regulatory noncoding transcription [23], or similar phenomena, could be responsible. Interestingly, we find nucleosomes at telomere 3L, but not at HMR or HML, are hypermethylated in alpha factor arrest (Figure S4), potentially secondary to the reported phosphorylation of Sir3 in response to MAPK signaling [24],[25]. Subtelomeric nucleosomes also lose their excess methylation during S phase, suggesting that alpha factor arrest may provide a physiological system to study the cell cycle dependence of heterochromatin maintenance and induction [26]–[28]. Whatever the physiological role of H3K4 methylation at genes whose mRNAs are not upregulated, it is important to note that this methylation is erased just as is the methylation associated with gene activity (see below).

### H3K4me3 Erasure Occurs within One Cell Cycle

**Figure 4:**
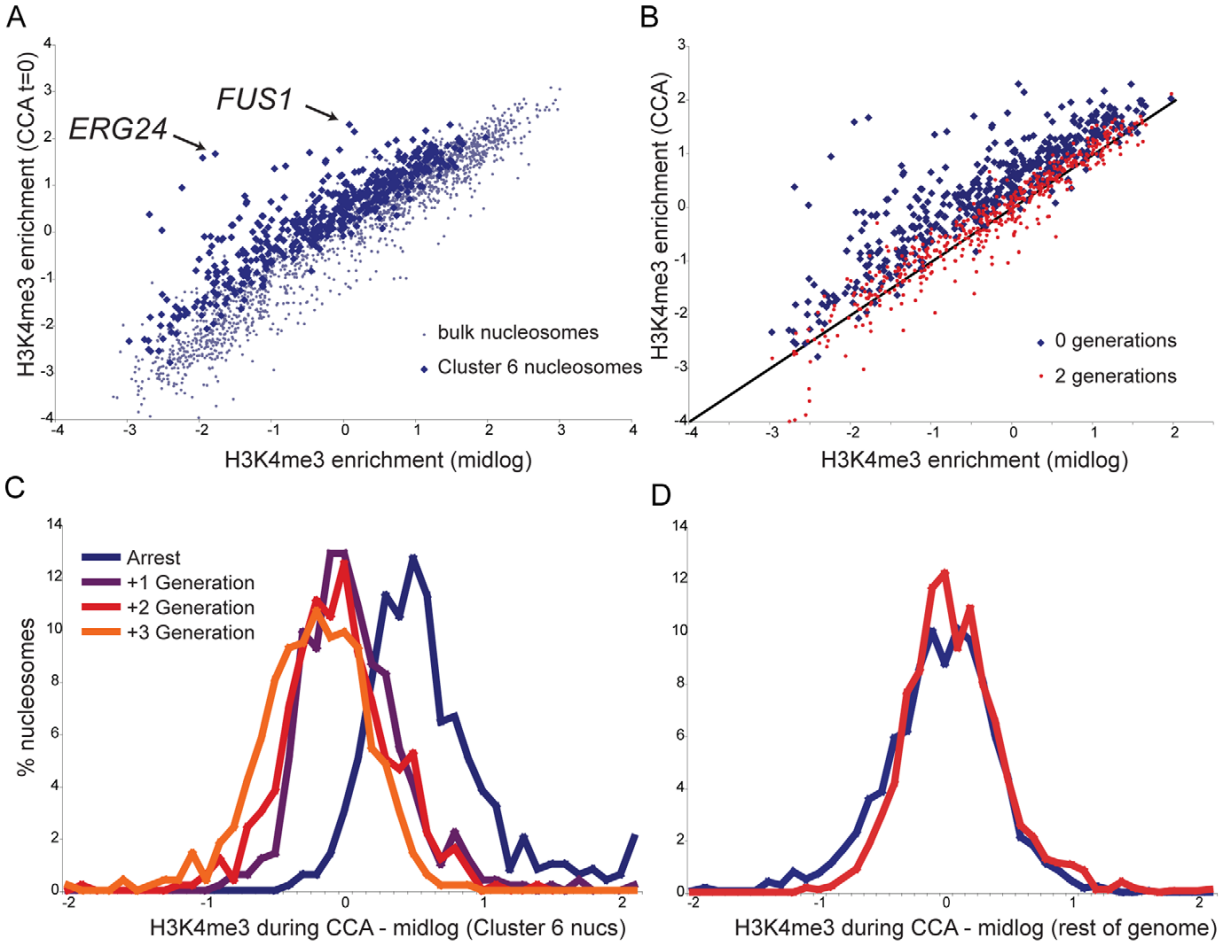
Kinetics of H3K4me3 loss during CCA. (A) Cluster 6 nucleosomes are more methylated during alpha arrest than during midlog growth. Scatterplot of H3K4me3 levels per nucleosome during midlog growth [6] (x axis) against levels during alpha arrest (y axis). Blue dots indicate the 473 Cluster 6 nucleosomes, grey dots indicate remaining nucleosomes. (B) Cluster 6 nucleosomes, midlog levels (x axis) versus levels during alpha arrest (y axis, blue dots), or two generations later (red dots). (C) Histogram of differences between midlog H3K4me3 levels and H3K4me3 levels at varying times during the CCA time course, for Cluster 6 nucleosomes. Most methylation has returned to midlog levels within one generation. (D) As in (C), for 1815 bulk (non-Cluster 6) nucleosomes.

Average H3K4me3 profiles of the nucleosomes matching Cluster 6 for each time course show a marked drop in S phase (Figure 3D), but the average quantitative level of this drop is less than two-fold. Of course, this represents the mean of many different nucleosomal profiles. We investigated H3K4me3 loss in more detail by comparing H3K4me3 levels per nucleosome in the CCA time course to H3K4me3 levels per nucleosome previously measured for midlog growth [6]. Figure 4A shows that Cluster 6 nucleosomes start out on average ∼40–50% more K4-trimethylated during CCA arrest than they are in midlog growth. Some notable outliers can be observed, such as 5′ nucleosomes over canonical alpha factor-inducible genes such as *FUS1* and *ERG24* (arrows), both of which are more than two-fold more methylated in alpha arrest than in midlog growth.

After two generations of growth, Cluster 6 nucleosomes reverted to baseline H3K4me3 levels (Figure 4B). How quickly are K4 methylation levels returned to baseline? The histogram of differences between H3K4me3 levels in midlog growth and levels during arrest for Cluster 6 nucleosomes reveals a distribution centered (in log_2_ space) on 0.5 (Figure 4C), as expected from Figure 4A. Notably, within one generation this distribution has shifted to an average of nearly zero, demonstrating that the majority of nucleosomes have reverted to midlog methylation levels within a single generation. The distribution changes little in the second or third generations after release, demonstrating that the majority of chromatin reprogramming occurred in the first generation. Importantly, the remainder of nucleosomes do not change appreciably during this time period (Figure 4D). Taken together, these data show that, by and large, H3K4 methylation patterns in one generation do not play an instructive role in establishment of chromatin structure in the next generation, as a single generation is largely sufficient to revert chromatin marks to new baseline levels.

### Kinetics of H3K4me3 Loss Implicate Replication in Erasure Process

**Figure 5:**
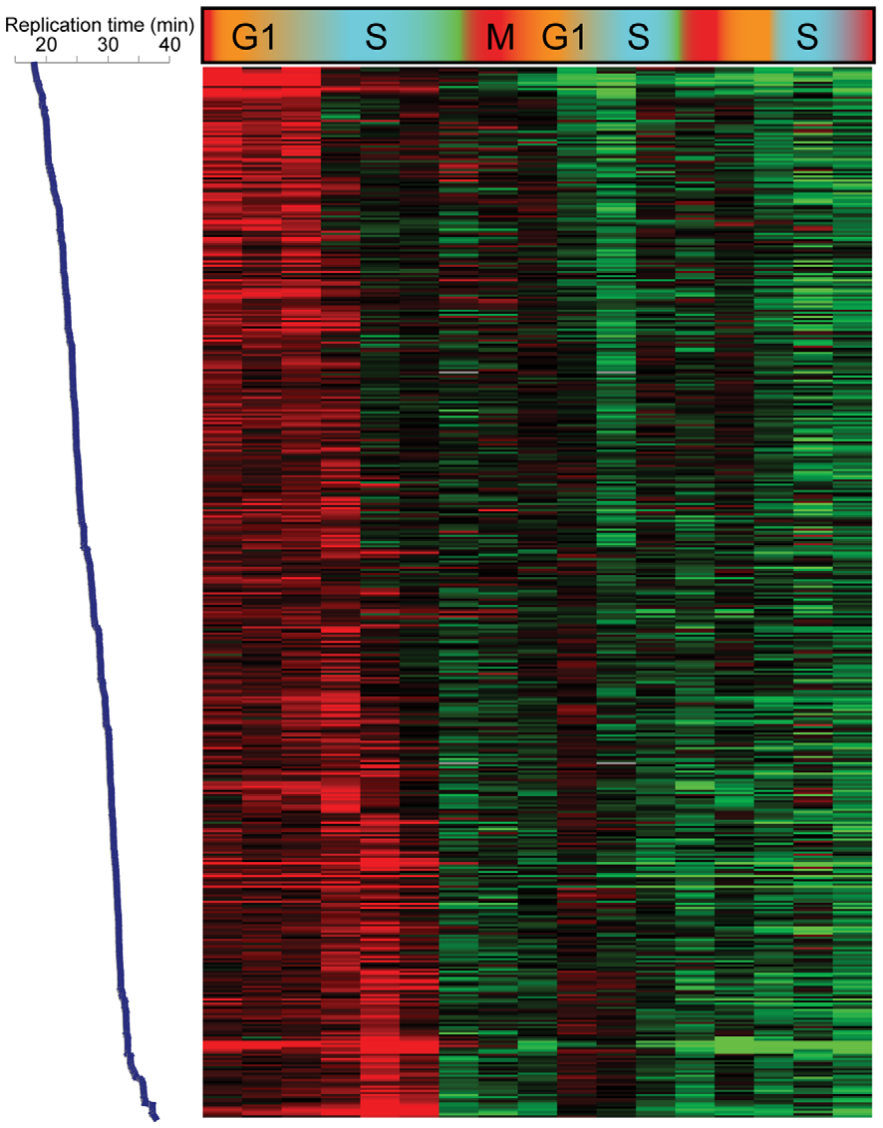
Replication-related loss of H3K4me3. Data for the 473 Cluster 6 nucleosomes from CCA, sorted by replication time from early (top) to late (bottom).

Is the S phase loss of H3K4me3 active or passive? A distinction can be made based on the extent of H3K4me3 loss. In principle, if an “old” maternal nucleosome re-associates with precisely the same location in one of the two daughter genomes that it occupied in the maternal genome, but the corresponding “new” nucleosome in the other genome is not methylated, then the level of H3K4me3 at that position will drop two-fold during S phase (seeMaterials and Methods for further discussion). A number of different mechanisms can give rise to a drop greater than two-fold, including but not limited to enzymatic demethylation [12],[15],[29], histone replacement [30],[31], tail cleavage [32],[33], or simple failure of some nucleosomes to re-associate with a daughter genome at the same locus they came from. We found that methylation levels at some of the most “over-methylated” nucleosomes did fall slightly more than two-fold across consecutive time points (for example, most nucleosomes over *FUS1* drop ∼2.5-fold in H3K4me3 at the beginning of S phase) (Figure S5), although they did not completely return to baseline after this drop. Methylation levels for these nucleosomes typically dropped most dramatically during S phase, then slowly decreased over the next cell cycle (see *FUS1* in Figure 3A and Figure S5 for an example). To further explore the potential role of replication in H3K4me3 loss, we aligned Cluster 6 nucleosomes by replication time [18], reasoning that simple failure to methylate newly-incorporated nucleosomes during S phase would manifest as S phase drops that correspond to replication timing. Indeed, for the CCA time course this is precisely what we see in Figure 5—K4 methylation is maintained at early-replicating nucleosomes until early S phase, while late-replicating nucleosomes lose H3K4me3 later in S phase. The correlation between replication timing and time of H3K4me3 loss was much more subtle during the poorer CCTS synchrony, but the same trend could be observed (Figure S6). These data are consistent with the simplest model for the majority of H3K4me3 loss – that newly-synthesized nucleosomes simply fail to be re-methylated in the absence of inducing stimuli.

### Genomic Replication Is Not Required for H3K4me3 Loss

**Figure 6:**
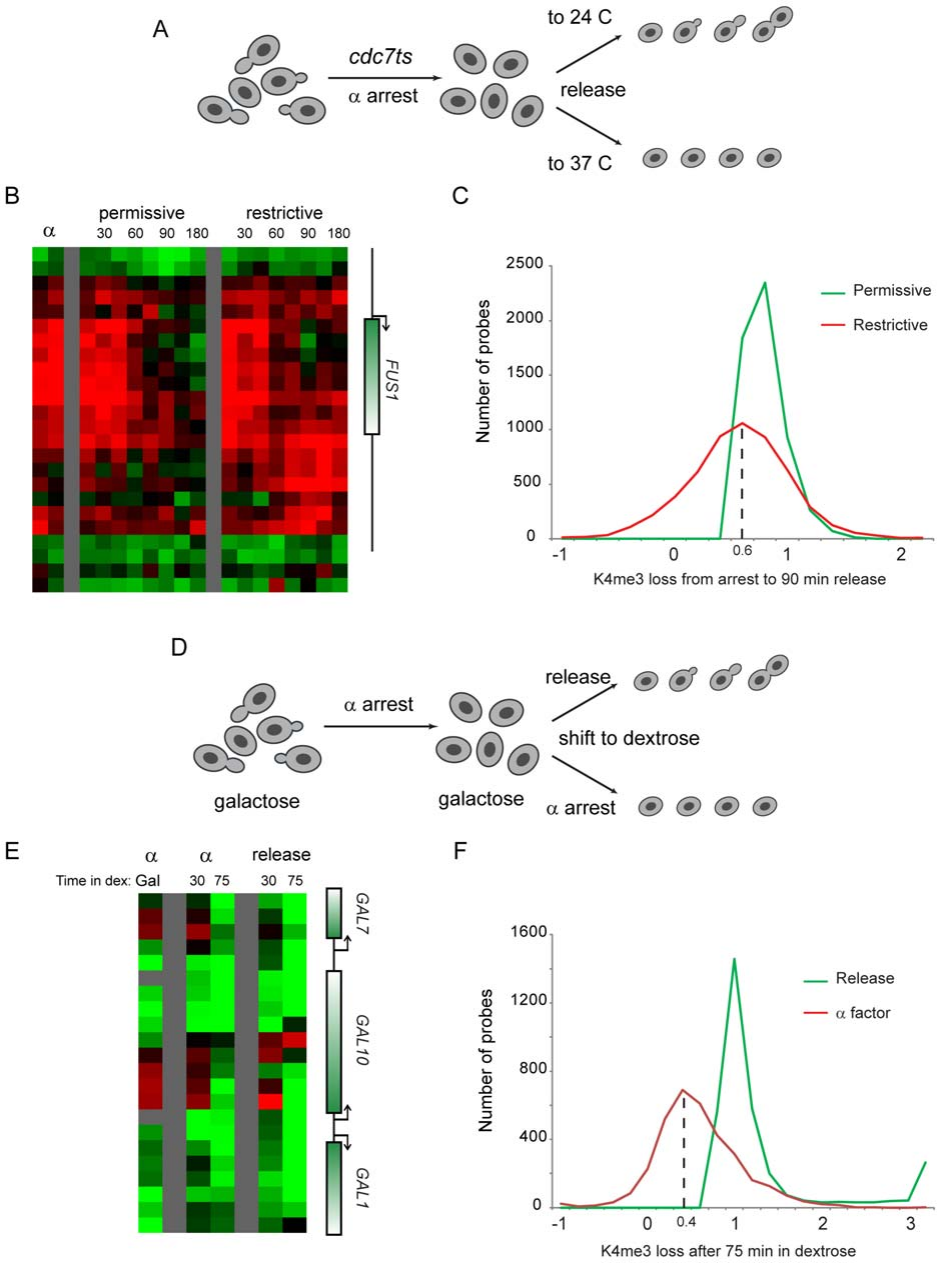
Replication is not required for loss of H3K4 methylation. (A) Schematic of experiment. *cdc7^ts^* yeast were arrested for four hours with alpha factor, then were released from alpha arrest to grow either at the *cdc7* permissive (24°C) or restrictive (37°C) temperature. H3K4me3 mapping was carried out on custom (B) and whole-genome (C) tiling microarrays. (B) *FUS1* methylation loss does not require genomic replication. Data from two time independent courses of alpha factor release are shown for 29 nucleosomes surrounding the *FUS1* gene. Time after release is shown in minutes above each time course. (C) Loci that lose methylation at the permissive temperature generally also lose methylation at the restrictive temperature. Probes with a methylation loss of over 40% from alpha factor arrest to 90 minutes release at the permissive temperature were selected. The extent of methylation change is histogrammed for permissive and restrictive temperature release (positive values indicate demethylation from arrest to the release time point)—x axis values are log(2). Probes that lose methylation at the permissive temperature generally also lose methylation at the restrictive temperature, albeit to a slightly lesser extent. We note that much of the variance at the permissive temperature can be ascribed to the heat shock of the restrictive temperature – most of the probes at the left of the histogram (ie not demethylated, or even overmethylated, at 37°C) are heat shock-induced genes, while many of the probes that lose methylation more at 37°C than at 24°C are associated with genes repressed during heat shock (analysis not shown). (D) Schematic of alternative system for replication-independent erasure. *bar1Δ* yeast were grown continuously in galactose, then arrested with alpha factor. Galactose-regulated genes were then shut off by shifting cells to dextrose, and were concomitantly either released from alpha factor arrest or maintained in alpha factor. (E) Methylation erasure over *GAL* genes does not require replication. Data from whole-genome tiling arrays shows high levels of H3K4me3 at the 5′ ends of *GAL1*, *GAL7* and *GAL10* when cells are arrested in alpha factor with galactose. Shift to dextrose results in loss of methylation whether cells are maintained in alpha factor or allowed to re-enter the cell cycle. (F) Loci that lose methylation during cell cycle release into dextrose generally also lose methylation when maintained in alpha factor. Analysis is similar to that shown in (C), but probes exhibiting higher methylation in alpha factor+galactose than in midlog+galactose (log2>0.75 difference) were eliminated to exclude confounding effects of erasure of these genes during release from alpha factor arrest. Furthermore, only probes with methylation decreases of 75% or more after 75 min of release into dextrose are shown.

We therefore tested the hypothesis that erasure of H3K4me3 was a result of passive dilution during replication. The Cdc7 kinase activates eukaryotic replication origins by phosphorylating Mcm proteins, and is required throughout S phase for origin firing [34],[35]. We synchronized *bar1Δ cdc7^ts^* yeast with alpha factor at the *cdc7^ts^* permissive temperature 24 C, then released them from alpha factor to either 24 C or the restrictive temperature 37 C (Figure 6A). FACS analysis confirmed the failure of these cells to replicate their genome at the restrictive temperature (Figure S7). Surprisingly, we found that *FUS1* H3K4 trimethylation was erased with identical kinetics in the presence and absence of genomic replication (Figure 6B). Interestingly, during release at the restrictive temperature an increase in H3K4 methylation is seen at the 3′ end of *FUS1*. Hybridization of H3K4me3 material from the 90 minute release time points to whole-genome tiling microarrays revealed that a subtle enhancement of 3′ methylation did occur broadly at the restrictive temperature, though it was not specific to loci undergoing erasure (see below).

Whole-genome data did confirm that loci erased at the permissive temperature were generally erased even without genomic replication, although this erasure was of lower magnitude (∼75%) in the absence of replication (Figure 6C, Figure S8). Interestingly, a recent study reported genome-wide mapping of H3K4me3 during meiosis in yeast [36], and argued that replication was not required for H3K4me3 erasure. Our reanalysis of this data revealed that H3K4me3 erasure during meiosis in that study was ∼70% efficient in the absence of replication (not shown), remarkably concordant with our value of ∼75%. We therefore identify a quantitative role for replication-dependent erasure/dilution in erasure of H3K4me3.

To further test the role of cell cycle progression in H3K4me3 erasure, we measured methylation levels genome-wide in galactose-grown yeast arrested in alpha factor (Figure 6D). Yeast were then shifted to dextrose-containing media to repress *GAL* and other carbon-related genes, and were either maintained in alpha factor to prevent cell cycle transit, or were released into the cell cycle. Consistent with the results obtained using the *cdc7^ts^* mutant, we found that erasure of H3K4 trimethylation over newly-repressed genes did not require cell cycle re-entry (Figure 6E and 6F). Again, the extent of H3K4me3 erasure was diminished in the absence of cell cycle transit (Figure 6F), indicating that dilution by replication likely does contribute quantitatively to H3K4me3 erasure.

It is important to note that there is substantial locus-to-locus variability in the extent to which replication contributes to H3K4me3 erasure (Figure 6C and 6F). Of course, much of this variation can be attributed to the relevant experimental manipulations – many of the loci that apparently fail to demethylate when *cdc7^ts^* yeast are shifted to 37 C (Figure 6C, left side of red curve) are associated with genes induced at high temperatures (not shown). In Figure 6F we attempted to control for this by eliminating probes associated with alpha factor-dependent methylation, so differences between continued alpha arrest and cell cycle release should not contribute to the variability in this case. To further explore locus-to-locus variability in the extent to which K4me3 can be erased via active mechanisms in the absence of genomic replication, we clustered patterns of K4 erasure in the genome-wide galactose-dextrose shift experiments (Figure S9). This analysis revealed that release-independent loss of methylation tended to occur at 5′ coding regions, whereas loss of methylation at 3′ ends of coding regions required release from alpha factor. These results suggest that active and passive erasure mechanisms may operate at distinct genomic loci – active mechanisms such as demethylation or histone replacement appear to preferentially operate at 5′ ends of coding regions, whereas excess H3K4me3 at 3′ ends of genes may be primarily cleared by replication.

### Demethylase Jhd2 Is Involved in H3K4me3 Demethylation

**Figure 7:**
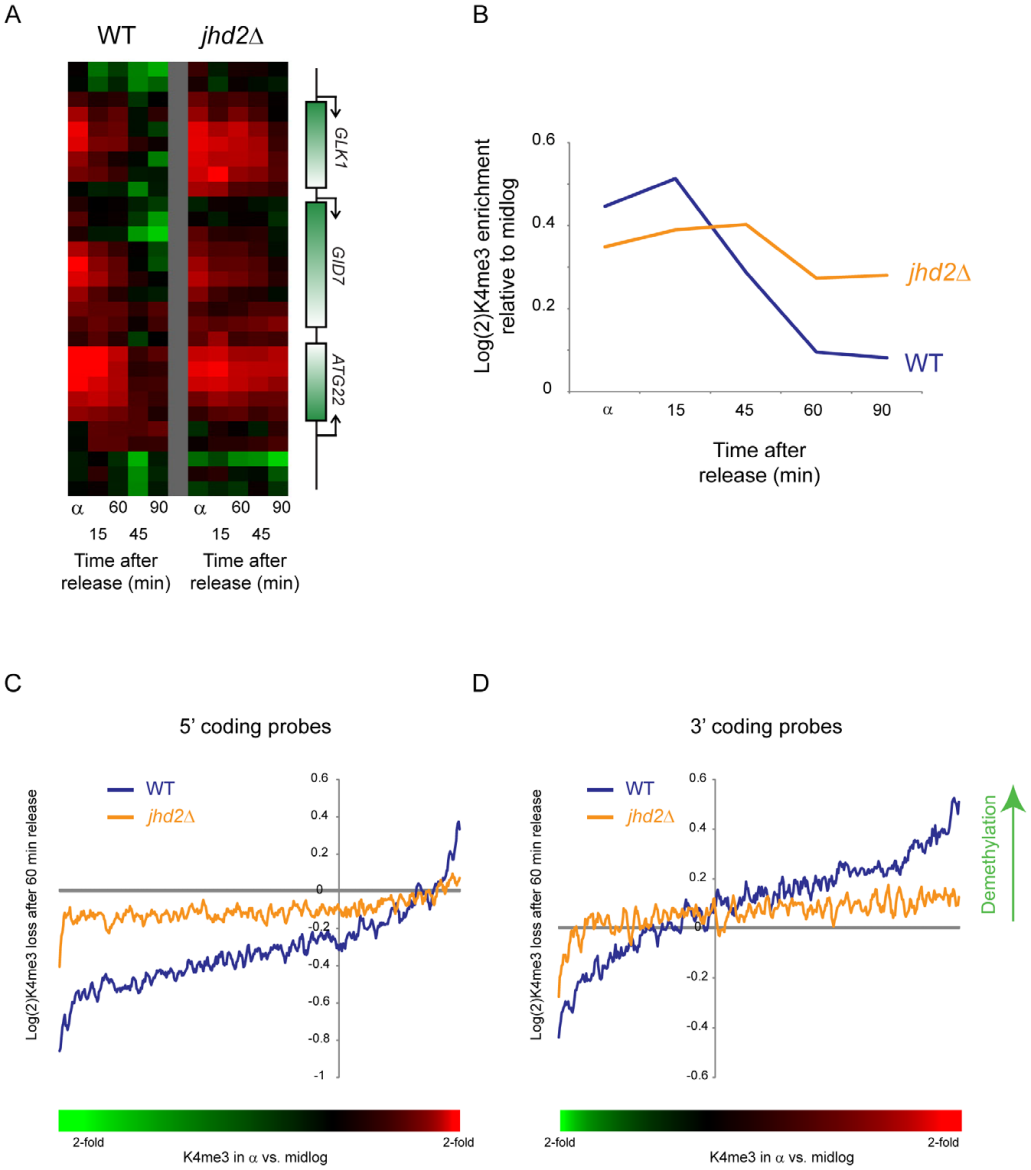
Delayed loss of H3K4me3 in *jhd2Δ* yeast. (A) H3K4me3 data are shown for a sample set of genomic loci during a short time course following alpha factor release. Data from wild type yeast are shown on the left, *jhd2Δ* on the right. (B) Global delay in H3K4me3 loss in *jhd2Δ*. Averaged data for early-replicating Cluster 6 nucleosomes from CCA are shown for wild-type and *jhd2Δ* yeast. (C,D) Inefficient H3K4me3 loss in *jhd2Δ* yeast. Whole-genome tiling microarrays were used to assay H3K4me3 levels during alpha arrest and after 60 minute release. 5′ coding probes (C) and 3′ coding probes (D) were ordered by the extent to which they are methylated in alpha factor arrest relative to midlog growth (bottom colorbar is used in place of x axis labels since axis is nonlinear. Red = hypermethylated in alpha factor, green = hypomethylated). Extent of methylation erasure after 60 minutes of release was calculated (erasure is positive), and a 50-probe running window average in presented. At both genic regions wild-type yeast erased H3K4me3 more efficiently than did *jhd2Δ* yeast, although in both cases methylation levels shifted away from the alpha arrest state back towards midlog levels (ie probes hypermethylated in alpha factor were demethylated upon release, hypomethylated probes were remethylated). Interestingly, there was a global baseline methylation loss at the 3′ ends of genes and a global methylation gain at 5′ ends—this was the result of a global shift in methylation patterns during alpha factor arrest—see Figure S12.

What is the mechanism for active erasure of K4 methylation? Yeast encode one major H3K4me3 demethylase, *JHD2*/*KDM5* [12],[37],[38]. We deleted *JHD2* in a *bar1Δ* background, arrested these yeast in alpha factor, then released parent and *jhd2Δ* yeast into the cell cycle. H3K4me3 loss was delayed in *jhd2Δ* yeast after release from alpha factor arrest (Figure 7A and 7B). This was not an artifact of a change in the kinetics of genomic replication, as FACS profiles of synchronized wild-type and *jhd2Δ* yeast were indistinguishable (Figure S10). Profiling of all three methylation states of H3K4 during a CCTS time course also provided circumstantial evidence for demethylation in H3K4me3 loss: the decrease in H3K4me3 at cluster 6 nucleosomes was followed by progressive peaks of di- and mono-methylation of H3K4 (Figure S11), as previously observed during a time course of heterochromatin establishment in yeast [15].

To further explore the role of Jhd2 in H3K4 demethylation, we compared genome-wide H3K4me3 profiles from wild-type and *jhd2Δ* yeast in alpha factor arrest and after 60 minutes of release. As shown in Figure 7C and 7D (and Figure S12), probes exhibiting excess H3K4 trimethylation in alpha factor arrest were demethylated in wild-type yeast much more efficiently than in *jhd2Δ*. These results together implicate enzymatic demethylation in H3K4me3 loss upon gene repression.

## Discussion

### H3K4me3 Is Globally Erased upon Removal of an Activating Stimulus

Previous studies on the *GAL* genes and on heterochromatic loci indicated that H3K4me3 is erased upon repression of the genes in question [1],[12],[15]. Here, we extended these studies to the whole genome in three different signaling contexts – alpha factor treatment, heat shock, and galactose. We found that upon release from these signaling regimes H3K4me3 levels fall completely to midlog baseline levels within 2 generations, with most demethylation occurring within the first generation. These results clearly demonstrate that typical active chromatin states, as characterized by levels of the long-lived activation mark H3K4me3, are not epigenetically maintained at genes after removal of an inducing stimulus. In other studies we have mapped other transcription-related histone modifications such H3K14ac and H2AK7ac (not shown), finding that they are erased within minutes of the condition shift, consistent with prior reports [10]. These results demonstrate that typical environmentally-responsive chromatin states are not epigenetically heritable *per se*, but are erased upon removal of activating stimulus, and therefore their maintenance requires active reestablishment.

### Cell Cycle Dynamics of H3K4 Methylation

An unanticipated aspect of H3K4 methylation loss at repressed genes is its occurrence during S phase. The greatest drop in K4me3 levels at newly-repressed genes in both alpha factor-synchronized and *cdc28-13*-synchronized yeast occurred during S phase. Furthermore, we found that H3K4me3 loss occurred early during S phase at early-replicating genomic loci, and occurred later at late-replicating regions (Figure 5). We also found that loci with the greatest levels of H3K4me3 relative to baseline (such as *FUS1* during alpha factor arrest—Figure S5) did not completely return to baseline during the first S phase, instead falling little over 2-fold at this stage. These results together strongly suggested a role for genomic replication, and resulting incorporation of unmethylated histone H3 at one of two daughter loci, in erasure of old active chromatin marks. Evidence for a general role for replication in methylation dynamics comes from Figure 1—even loci that are not “overmethylated” during arrest transiently lose methylation immediately after replication, suggesting that the role for replication in H3K4me3 loss is not specific to genes that are being repressed.

Surprisingly, direct tests of the role for replication in H3K4me3 loss revealed that replication is not absolutely required for H3K4me3 erasure at canonical targets of alpha factor signaling (*FUS1*) or galactose (*GAL* genes)—see Figure 6. We did find a quantitative role for replication in K4me3 erasure, with a global ∼25–40% decrease in H3K4 “demethylation” under two independent non-replicating conditions. Interestingly, re-analysis of H3K4me3 erasure during meiosis [36] identifies a similar 25% decrease in methylation loss when genomic replication is blocked in this system. These results are also in quantitative agreement with a single-locus study in *S. cerevisiae* in which mating loci were excised onto nonreplicating circles before induction of heterochromatin [15] – here too H3K4me3 is erased in the absence of replication, but H3K4me3 levels were ∼30% higher in the absence of replication than in the native (replicating) context. Together, these results have implications for the establishment of epigenetic silencing states, which appears to require S phase passage [26],[39], but may not require replication [27],[28],[40].

We interpret these results together to indicate that genomic replication does result in a general two-fold methylation erasure (even at loci that are not being actively demethylated—see Figure 1), but this dilution is one of at least two mechanisms contributing to H3K4me3 loss along with enzymatic demethylation. One simple prediction of this model is that *jhd2Δ* yeast should eventually erase ancestral H3K4me3 states via replication alone, a prediction borne out by the fact that H3K4me3 mapping in these mutants does not show signatures of ancestral exposures to cold shock (the refrigerator) or diauxic shift (overnight culture).

The coincidence of the major loss of methylation with S-phase in this context therefore suggests that enzymatic demethylation is typically gradual (Figure S5), and thus for a given time point in a synchronized population the two-fold loss secondary to replication dominates the overall methylation decrease at that time point. Interestingly, active demethylation occurred independently of replication at 5′ ends of genes, but clearance of excess methylation at 3′ ends of genes appeared to require replication (Figure 6A, Figure S9), suggesting that active demethylation is specifically targeted to the 5′ end of the gene where most H3K4me3 normally occurs [6],[7].

In this framework, one pair of results still remains to be reconciled. Specifically, a dramatic H3K4 demethylation event occurs after a significant (∼45 minute) delay even in the absence of genomic replication in *cdc7^ts^* yeast (Figure 6B), which might suggest an S phase event (independent of replication) resulting in enhanced demethylase activity. However, demethylation at *GAL* genes upon carbon source shift occurred even when yeast were maintained in alpha factor, indicating that at least some H3K4me3 erasure occurs during G1 arrest, albeit after a long time (Figure 6E). Thus, we do not currently understand the reason for the delay that typically occurs between removal of an activating stimulus and the major demethylation event.

### H3K4me3 and Epigenetic Inheritance

The idea that chromatin states are heritable, while widely cited, is still a matter of debate [41]. A major piece of evidence for the heritability of chromatin states is the genetic requirement for histone modifying enzymes in a number of epigenetic inheritance systems – in *S. cerevisiae* and *P. falciparum*, variegated repression of subtelomeric genes requires histone deacetylases [13], [42]–[44], and subtelomeric loci in the epigenetic OFF state are packaged into a distinctive chromatin structure that differs significantly from the packaging state of the epigenetically ON state [45],[46]. During metazoan development, memory of repressed states requires Polycomb proteins, which are involved in methylation of H3K27, while memory of active states is associated with methylation of histone H3 lysine 4 by Trithorax group proteins [2].

However, in yeast a handful of studies on *GAL* and *SUC* genes indicated that H3K4me3 was lost upon dextrose repression [1],[12], and similar results were found even at the epigenetically-regulated heterochromatic loci upon induction of heterochromatin [15]. The kinetics of H3K4me3 loss in these studies suggested that erasure was a consequence of active demethylation, and deletion of *JHD2*/*KDM5* implicated this demethylase in the process. Our results with *jhd2Δ* mutants confirm a global role for Jhd2 in accelerating loss of H3K4 methylation, although it is important to note that in the absence of Jhd2, H3K4 methylation still could be erased, but only after an extended delay (Figure 7A).

Thus, these results demonstrate that *most* chromatin states are unlikely to be heritable per se, and if chromatin states ever are maintained through cell division this is likely to require specific maintenance machinery that operates at specialized genomic loci (for example, the Hox loci in metazoans). We hypothesize that most other environmentally-responsive chromatin states will also be erased and require active reestablishment for their maintenance. Intriguingly, recent results in *C. elegans* show that enzymatic erasure of H3K4me3 patterns is required for correct functioning of the germline [47], consistent with a general role for demethylases in erasing inappropriate ancestral chromatin states. In this view, the majority of chromatin modifications do not generate epigenetic signals [41].

## Materials and Methods

### Strain Construction

*jhd2Δ* (*MAT* ***a*** *ura3Δ leu2Δ his3Δ met15Δ bar1Δ::HIS5 jhd2Δ::KanR*):

A PCR fragment containing the Kanamycin resistance gene and ends complementary to the *JHD2* locus (primer sequences: 5′ATGGAGGAAATTCCTGCCCTGTATCCAACGGAACAAGACCAGCTGAAGCTTCGTACGC and 5′CTATCTATCTAACTTAACACCAACTTGCTTTATTAAAGAGGGCGCGAGGATCGTAATAAG; pCM224 plasmid as template) was transformed into the yeast JOY1 strain (*MAT* ***a*** *ura3Δ leu2Δ his3Δ met15Δ bar1Δ::HIS5*). Stable transformants were selected on G418 plates and correct integration into the *JHD2* locus was confirmed by PCR (*JHD2* locus primers: 5′TCATGGAGGAAATTCCTGCCCTGTATCCAA and CTATCTATCTAACTTAACACCAACTTGCTTTATTAAAGAG; KanR-specific primers: 5′AGGAATCGAATGCAACCGGC and 5′TATGGGTATAAATGGGCTCGCG).

RM14-3a (*MAT* **a** *cdc7-1 bar1 ura3-52 trp1-289 leu2-3,112 his6*) [34] was obtained from Manolis Papamichos-Chronakis.

### Yeast Culture

For the CCA time course, 18 2L flasks each of 450 mL BY4741 *bar1Δ* cells were grown in YPD as three groups of 6 flasks, to an A_600_ OD of 0.25 (Group 1, time points 100–150 min. at 10 min. intervals), 0.4 (Group 2, time points 40–90 min. at 10 min. intervals), and 0.7 (Group 3, 0–30 min. at 5–10 min. intervals). Cells were arrested for 3 hours with the addition of 450 µL of 1 mg/ml alpha factor, then filtered with 0.22 µM Whatman filter, washed with water, and transferred to a prewarmed flask containing 450 ml YPD and 22.5 mg pronase. Each 450 mL culture was stopped with 1% formaldehyde at the appropriate time. For the CCTS time course, cells were cultured as described in [17]. For each time point, 50 ml of cell culture were spun and flash frozen on liquid nitrogen for mRNA isolation. With the remainder, 37% formaldehyde was added to a 1% final concentration, and the cells were incubated for 15 minutes at room temperature, shaking, at 200 rpm. 2.5 M glycine was added to a final concentration of 125 mM to quench the formaldehyde. The cells were transferred to a 500 mL centrifuge jar on ice and then let stand until groups of 6 time points had accumulated. The cells were spun down at 3000×g for 5 minutes at 4°C and washed once with 50 mL of room temperature MilliQ water. Subsequent procedures were performed in batches of 6 time points.

For CCA repeat with wt and *cdc7^ts^* yeast (Figure 6A–6C), two flasks of cells were grown to OD 0.2 at 24 C and arrested for 4hrs with 1 µg/ml alpha factor. Each culture was released from arrest by filtering, and cells were added to media prewarmed to either 24 C or 37 C to which 20 µg/ml pronase (SIGMA) was added. 100ml were taken from the common culture at indicated times and fixed in 2% formaldehyde. Figure 6B shows results from two interleaved time courses at each temperature.

For CCA repeat with galactose to dextrose media switch (Figure 6D–6F), one flask of *bar1Δ* cells (JOY1 strain) was grown in YPgalactose to OD 0.3 at 30C and arrested for 3hrs with 1 µg/ml alpha factor. The culture was then filtered in two batches and each filter was put into YPdextrose media either with 1 µg/ml alpha factor or with 20 µg/ml pronase (SIGMA). 100ml were taken from the two cultures at indicated times and fixed in 2% formaldehyde.

For CCA repeat with wt and *jhd2Δ* yeast (Figure 7), cells were grown to OD 0.2 and arrested for 3hrs with 1 µg/ml alpha factor. The whole culture (750ml) was released from arrest by filtering and addition of 20 µg/ml pronase and 100ml were taken from the common culture at indicated times and fixed in 2% formaldehyde.

### Chromatin Mapping

H3K4me3 levels were determined essentially as described previously [6],[17]. For both CCA and CCTS time courses, cells were first digested with micrococcal nuclease and immunoprecipitated, except with the following antibodies: (CCA Ab: 4 µL anti-H3K4Me3 (polyclonal, Abcam #Ab8580); CCTS and CCA repeat Ab: 5 µL anti-H3K4Me3 mAb clone MC315 (Upstate #05-745; affinity purified; this is no longer being offered by Upstate)). Antibody specificity information is available at the suppliers' websites. Following the ChIP, proteins were degraded and DNA purified as described in [17], and linear amplification was carried out as in Liu et al [6],[48]. For nucleosome occupancy profiling, amplified nucleosomal material from a given time point was competitively hybridized against pooled nucleosomal DNA from the entire time course, resulting in relative occupancy measures over the two cell cycle time courses.

Protocol is available online at:

http://www.broad.harvard.edu/chembio/lab_schreiber/pubs/protocols/IVT_Supplement/supplement.html.

### Microarray Hybridization

3 µg of aRNA produced from the linear amplification were used to label probe via the amino-allyl method, and microarrays were hybridized, scanned, and processed as described previously [6],[17]. CCA experiments were hybridized as ChIP against input [6], whereas CCTS experiments were hybridized as ChIP or nucleosomal input at time = t against a reference pool composed of all time points in the time course [17]. Data for both time courses are zero-centered per nucleosome (except for analysis in Figure 4) to make them comparable. For Figure 6, Figure 7, Figure S7, Figure S8, and Figure S12, material from the indicated strains/conditions was hybridized to Agilent 4X44K whole genome microarrays.

Data have been submitted to GEO, accession #GSE14565.

### Data Analysis

For Figure 1 and Figure 5, replication timing data was taken from Yabuki et al [18], mapped to our array as in Kaplan et al [17]. For Figure 2, H3K4me3 data for CCA and CCTS were concatenated and subjected to k-means clustering with k = 6. Average time course profiles for CCA and CCTS were generated from original Cluster 6 nucleosomes, and all CCA (or CCTS, respectively) nucleosomes with a correlation> = 0.5 to this average were added to the “Cluster 6” set. In Figure 3B nucleosomes from CCA and CCTS Cluster 6 sets were combined, are sorted by the difference in correlation to the original Cluster 6 CCA or CCTS average profile. For Figure 3C, each Cluster 6 nucleosome was associated with a nearby gene [6], and the median expression previously reported during a heat shock [21] or alpha factor [22] time course was calculated. Expression data is presented and average +/− standard error of the mean for each group of nucleosomes.

### Comment on Quantitative Extent of Demethylation

When discussing the mechanism of loss of H3K4me3 (active vs. passive) in the text, we ignore the presence of two H3 molecules in a nucleosome. There are two reasons for this. First, it is currently unknown how ChIP efficiency will vary for nucleosomes carrying one vs. two H3K4me3 marks, and we suspect these efficiencies might be quite similar. The possibility that ChIP is efficient for both mono- and di-modified nucleosomes simply implies that our measurements of H3K4me3 decreases could be underestimates. Second, the majority of evidence suggests that H3/H4 tetramers do not split during replication. The lack of splitting would lead to the expected ceiling of two-fold loss of methylation via dilution. These considerations do not affect any conclusions of the paper, but are worth keeping in mind when evaluating mechanistic models to be tested against histone modification mapping data. Of course since we directly test replication and enzymatic demethylation here, we only include this issue as a supplemental note for interested readers.

### FACS Analysis

During the CCA *cdc7^ts^* and *jhd2Δ* experiments in Figure 6 and Figure 7, 1 ml cell aliquots were taken at indicated times, cells were pelleted and fixed in 5ml 70% ethanol and refrigerated overnight. Cells were then pelleted and washed twice with PBS, resuspended in 300ul PBS/0.08mg/ml RNAseA (Qiagen). A 2hrs 37C incubation was followed by a 30sec sonication in a Bronson cup sonicator (setting 3, constant duty cycle). Pelleted cells were than resuspended in 400 µl PBS/1 µM SYTOX green (Invitrogen) and analyzed with a Beckton Dickson FACS machine.

## Supporting Information

Figure S1 Gene expression profiling demonstrates good synchrony. Data for ∼800 cell cycle-regulated genes defined by Spellman, et al. are arranged by phase of peak expression. Data from Spellman are shown, followed by our data from CCA (alpha factor arrest/release) and CCTS (arrest/release of *cdc28-13* by temperature shift) as indicated. (Spellman PT, Sherlock G, Zhang MQ, Iyer VR, Anders K, et al. (1998) Comprehensive identification of cell cycle-regulated genes of the yeast Saccharomyces cerevisiae by microarray hybridization. Mol Biol Cell 9: 3273–3297.)
(1.89 MB PDF)

Figure S2 Nucleosome occupancy and H3K4me3 versus replication timing during CCTS. Enrichment of nucleosome occupancy (A) and H3K4me3 (B) are plotted for 10% bins of nucleosomes over the course of CCTS, as in Figure 1B and 1C.
(0.24 MB PDF)

Figure S3 Schematic interpretation of Cluster 6 nucleosomes. (A) Cell cycle synchrony involves a condition shift. Methods of cell cycle synchrony typically involve growth under some condition for synchronization (here, either alpha factor for CCA, or high temperature for CCTS), with release requiring a shift into a new growth media. (B) Schematic for a gene highly transcribed during the arrest, but not after release. How do old “active state” nucleosomes get removed/erased/disassembled when the activating stimulus is removed?
(0.23 MB PDF)

Figure S4 Subtelomeric nucleosomes are methylated during alpha factor arrest, and are demethylated along with other Cluster 6 nucleosomes. Data for 9 nucleosomes at TEL3L shown as in Figure 1A.
(0.25 MB PDF)

Figure S5 H3K4me3 removal at *FUS1* exhibits a rapid and a slow phase. H3K4me3 enrichment (relative to [6]) is plotted for the 8 nucleosomes over *FUS1*, and for the average of these 8. Note that the average S phase drop (from 10 to 15 minutes) in H3K4me3 is ∼1.5 in log(2) units, or ∼2.9-fold.
(0.23 MB PDF)

Figure S6 H3K4me3 loss versus replication timing during CCTS. (A) Cluster 6 nucleosomes are sorted by replication timing and plot is as in Figure 4A, but for CCTS. (B) CCTS data for the 20% earliest (blue) or latest (green) replicating nucleosomes was averaged, and plotted over time. Note the subtle delay in H3K4me3 loss at later-replicating nucleosomes.
(0.50 MB PDF)

Figure S7 *cdc7^ts^* yeast do not replicate their genomes at the restrictive temperature. FACS analysis of two time courses each of *cdc7^ts^* yeast released from alpha factor arrest to 24 C (A) or 37 C (B) for varying times.
(0.97 MB PDF)

Figure S8 Global demethylation is not replication-dependent. H3K4me3 material from *cdc7^ts^* yeast arrested in alpha factor, or released for 90 minutes at 24°C or 37°C, was hybridized against whole-genome tiling microarrays. (A) Broad changes in methylation upon alpha factor release are consistent in the presence or absence of genomic replication. Demethylation was calculated as the difference between a probe's K4me3 enrichment after 90 min release and that probe's K4me3 enrichment during alpha factor arrest. Data are sorted by extent of methylation change at the permissive temperature, and a 100 probe running window average is shown for the two release temperatures.
(0.32 MB PDF)

Figure S9 Efficient S phase-independent H3K4me3 erasure occurs preferentially at 5′ ends of coding regions. (A) Whole-genome microarray data for wild-type cells grown as indicated in Figure 6D. Probes exhibiting above-average H3K4me3 levels during midlog growth, but which did not gain H3K4me3 during alpha factor arrest, were subjected to k-means clustering with k = 4. Cluster 3 probes indicate probes that lose H3K4me3 upon addition of dextrose, independent of alpha factor release (compare rightmost two columns). Conversely, Cluster 1, 2, and 4 exhibit greater demethylation upon alpha factor release. (B) 3′ coding regions exhibit release-dependent demethylation. Probes from Clusters 1–4 were ordered according to distance from transcription start site (x axis), and average H3K4me3 loss 75 minutes after dextrose addition is shown for continued arrest (α) or concomitant release. Note that Cluster 3 nucleosomes (green) exhibit release-*independent* 5′ demethylation, whereas all clusters showed release-*dependent* 3′ demethylation.
(0.33 MB PDF)

Figure S10 Jhd2 does not affect cell cycle synchrony. FACS analysis of wild-type (A) and *jhd2Δ* (B) yeast during alpha factor arrest, and at varying times after release.
(0.26 MB PDF)

Figure S11 Progressive demethylation at H3K4. Averaged data for H3K4me3, me2, and me1 are shown for Cluster 6 nucleosomes during a replicate of the CCTS time course.
(0.22 MB PDF)

Figure S12 Global changes in H3K4me3 during alpha factor arrest. (A) Coding region H3K4me3 flattens during alpha factor arrest. Distributions of H3K4me3/nucleosome were calculated for 5′ and 3′ probes during midlog and alpha factor arrest (indicated), showing that 3′ probes, which are normally hypomethylated become more methylated during arrest. (B) Alpha factor arrest results in H3K4me3 extension into coding regions. Averaged data for high, middle, and low expression level genes are shown with probes ordered according to distance into coding region. Note that during alpha factor arrest the normally 5′-biased H3K4me3 pattern extends further into coding regions, particularly at poorly-expressed genes. (C) Jhd2 is required to counteract alpha factor-dependent H3K4me3 flattening. As in (A), except all genomic probes are included in histograms. Note that both in wild-type and *jhd2Δ* yeast H3K4me3 distributions become more unimodal during alpha factor arrest, and this is reversed upon release in a Jhd2-dependent manner.
(0.31 MB PDF)

Table S1 Nucleosome occupancy and H3K4me3 data for CCA. Data are granularized by nucleosome.
(3.17 MB XLS)

Table S2 Nucleosome occupancy and H3K4me3 data for CCTS. Data are granularized by nucleosome.
(1.55 MB XLS)
