# Supplementary figures and images for: Replication and Active Demethylation Represent Partially Overlapping Mechanisms for Erasure of H3K4me3 in Budding Yeast
Source: PLoS Genet. 2010 Feb 5;6(2):e1000837. doi: 10.1371/journal.pgen.1000837 (PMC2816684; doi:10.1371/journal.pgen.1000837)

Figure S1

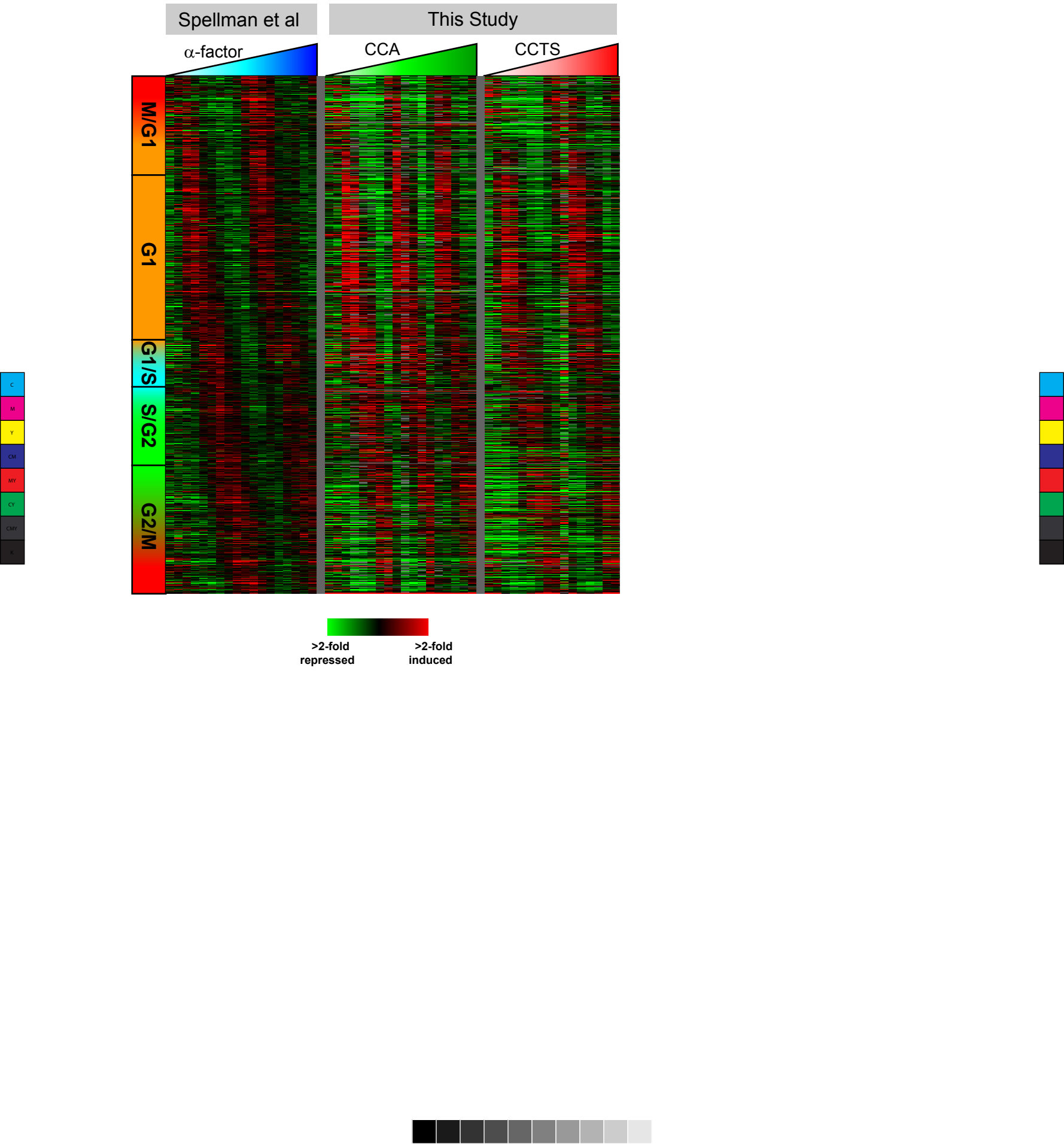

Supplement: Figure S1 — Gene expression profiling demonstrates good synchrony. Data for ∼800 cell cycle-regulated genes defined by Spellman, et al. are arranged by phase of peak expression. Data from Spellman are shown, followed by our data from CCA (alpha factor arrest/release) and CCTS (arrest/release of cdc28-13 by temperature shift) as indicated. (Spellman PT, Sherlock G, Zhang MQ, Iyer VR, Anders K, et al. (1998) Comprehensive identification of cell cycle-regulated genes of the yeast Saccharomyces cerevisiae by microarray hybridization. Mol Biol Cell 9: 3273–3297.) (1.89 MB PDF) [file pgen.1000837.s001.pdf]

Figure S2

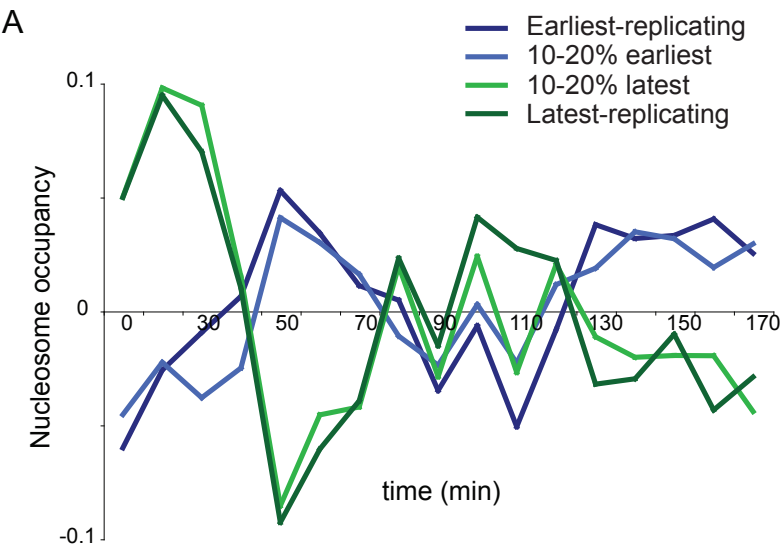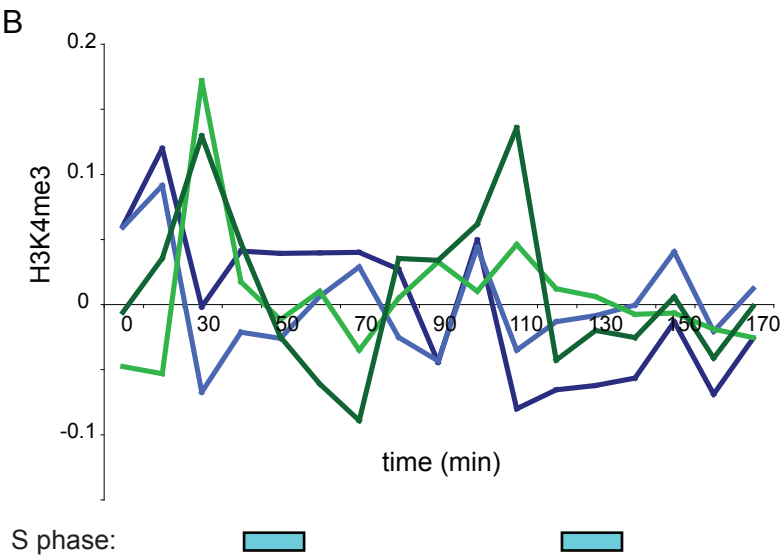

Supplement: Figure S2 — Nucleosome occupancy and H3K4me3 versus replication timing during CCTS. Enrichment of nucleosome occupancy (A) and H3K4me3 (B) are plotted for 10% bins of nucleosomes over the course of CCTS, as in Figure 1B and 1C. (0.24 MB PDF) [file pgen.1000837.s002.pdf]

Figure S3

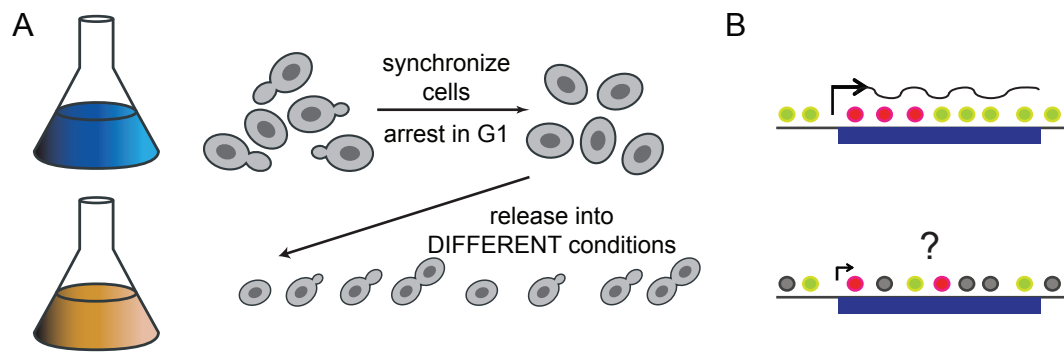

Supplement: Figure S3 — Schematic interpretation of Cluster 6 nucleosomes. (A) Cell cycle synchrony involves a condition shift. Methods of cell cycle synchrony typically involve growth under some condition for synchronization (here, either alpha factor for CCA, or high temperature for CCTS), with release requiring a shift into a new growth media. (B) Schematic for a gene highly transcribed during the arrest, but not after release. How do old “active state” nucleosomes get removed/erased/disassembled when the activating stimulus is removed? (0.23 MB PDF) [file pgen.1000837.s003.pdf]

Figure S4

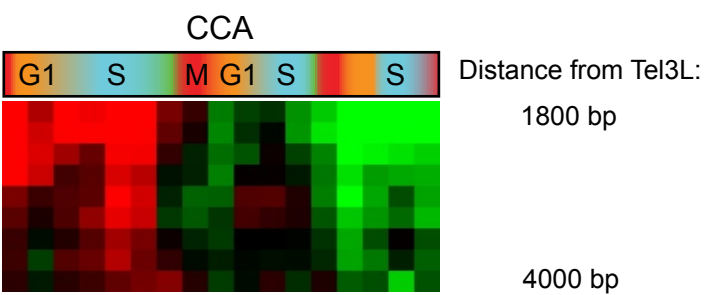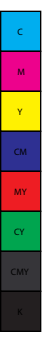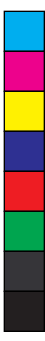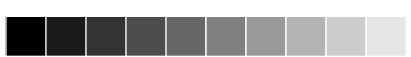

Supplement: Figure S4 — Subtelomeric nucleosomes are methylated during alpha factor arrest, and are demethylated along with other Cluster 6 nucleosomes. Data for 9 nucleosomes at TEL3L shown as in Figure 1A. (0.25 MB PDF) [file pgen.1000837.s004.pdf]

Figure S5

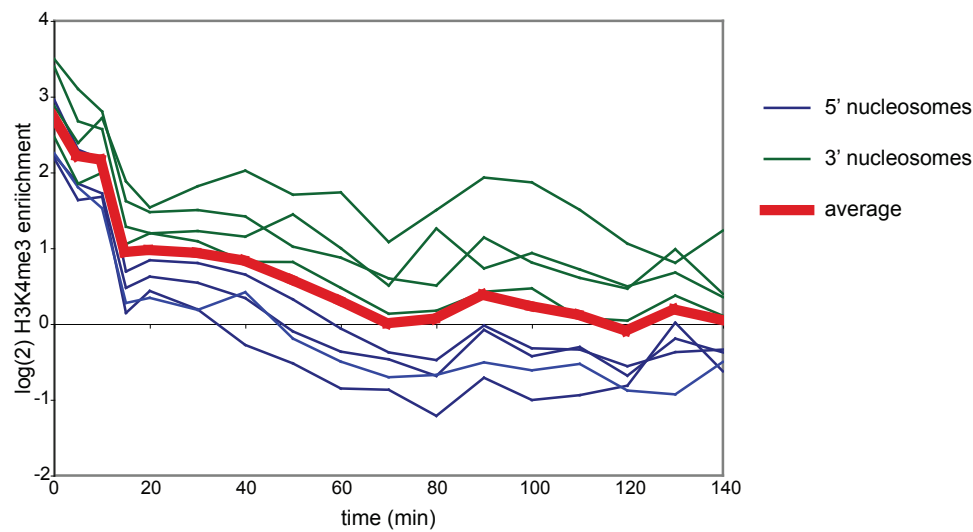

Supplement: Figure S5 — H3K4me3 removal at FUS1 exhibits a rapid and a slow phase. H3K4me3 enrichment (relative to [6]) is plotted for the 8 nucleosomes over FUS1, and for the average of these 8. Note that the average S phase drop (from 10 to 15 minutes) in H3K4me3 is ∼1.5 in log(2) units, or ∼2.9-fold. (0.23 MB PDF) [file pgen.1000837.s005.pdf]

Figure S6

A

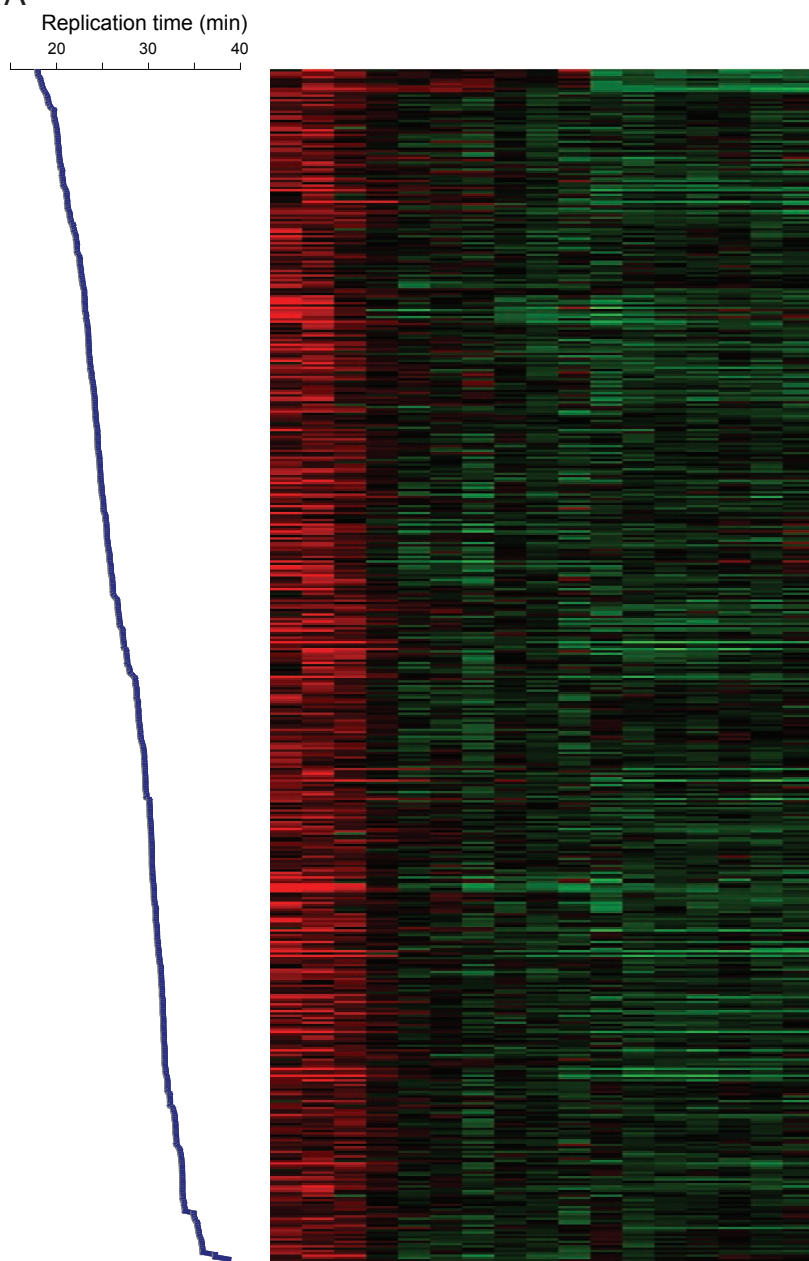

B

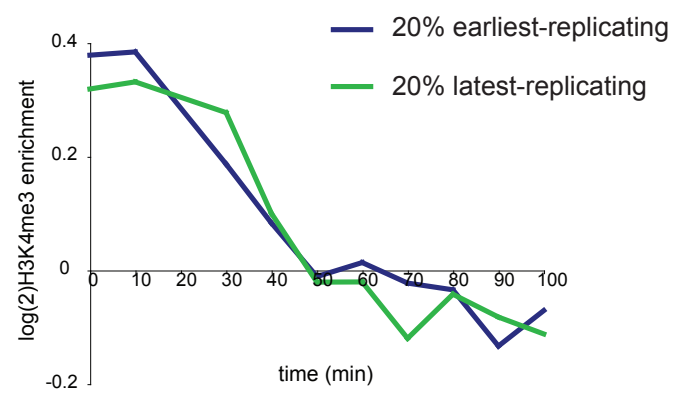

Supplement: Figure S6 — H3K4me3 loss versus replication timing during CCTS. (A) Cluster 6 nucleosomes are sorted by replication timing and plot is as in Figure 4A, but for CCTS. (B) CCTS data for the 20% earliest (blue) or latest (green) replicating nucleosomes was averaged, and plotted over time. Note the subtle delay in H3K4me3 loss at later-replicating nucleosomes. (0.50 MB PDF) [file pgen.1000837.s006.pdf]

Figure S7

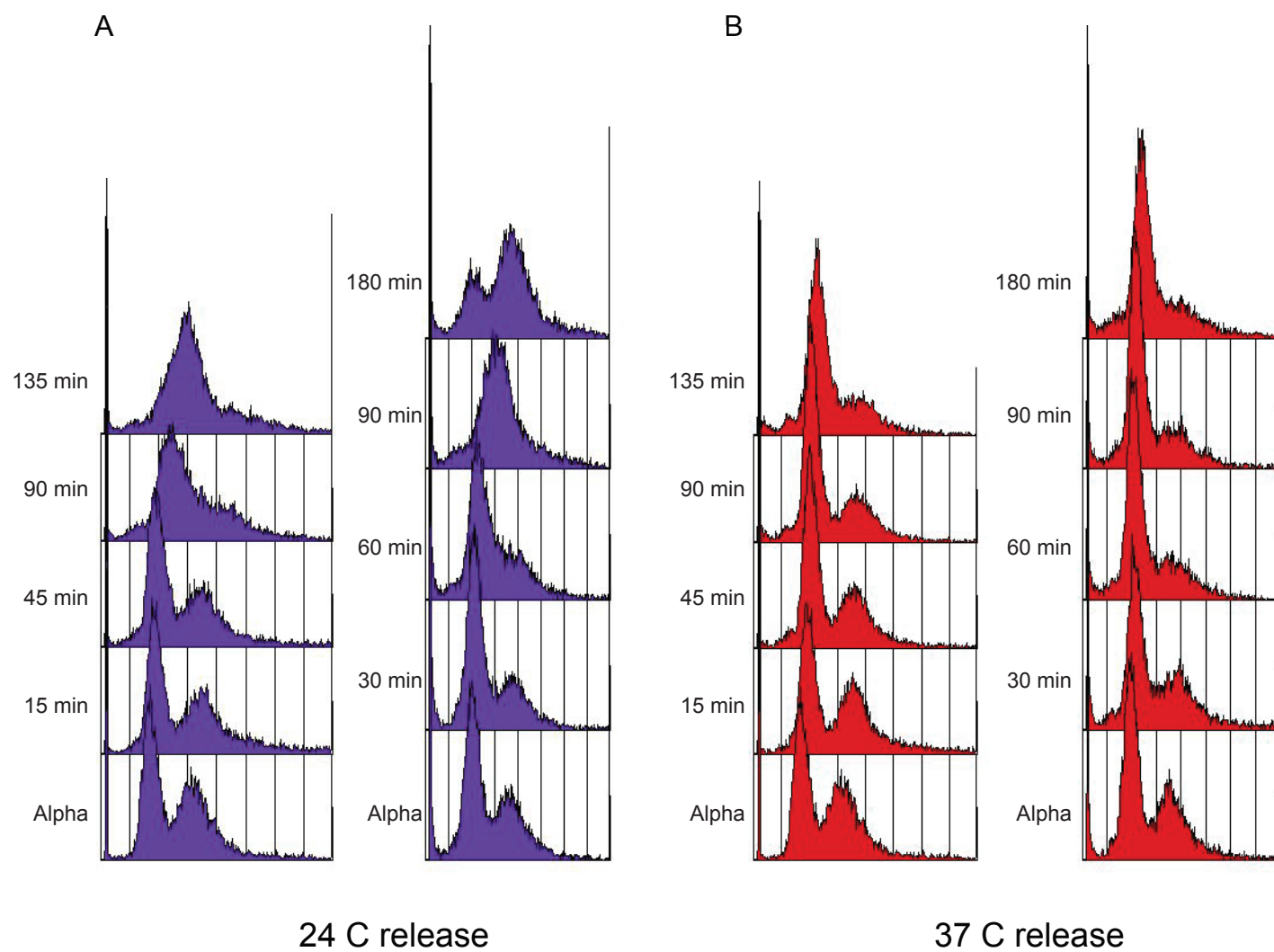

Supplement: Figure S7 — cdc7ts yeast do not replicate their genomes at the restrictive temperature. FACS analysis of two time courses each of cdc7ts yeast released from alpha factor arrest to 24 C (A) or 37 C (B) for varying times. (0.97 MB PDF) [file pgen.1000837.s007.pdf]

Figure S8

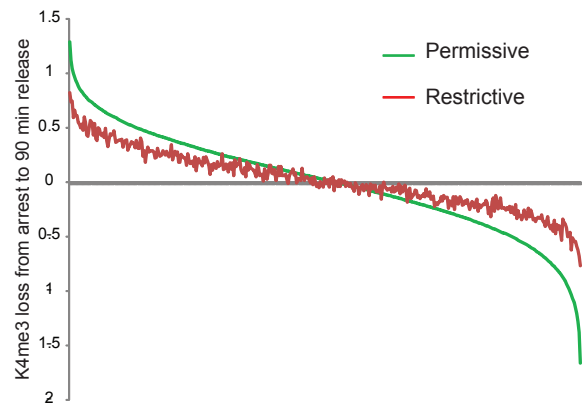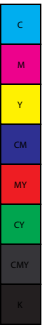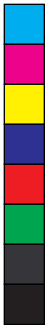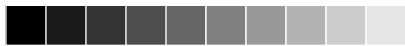

Supplement: Figure S8 — Global demethylation is not replication-dependent. H3K4me3 material from cdc7ts yeast arrested in alpha factor, or released for 90 minutes at 24°C or 37°C, was hybridized against whole-genome tiling microarrays. (A) Broad changes in methylation upon alpha factor release are consistent in the presence or absence of genomic replication. Demethylation was calculated as the difference between a probe's K4me3 enrichment after 90 min release and that probe's K4me3 enrichment during alpha factor arrest. Data are sorted by extent of methylation change at the permissive temperature, and a 100 probe running window average is shown for the two release temperatures. (0.32 MB PDF) [file pgen.1000837.s008.pdf]

Figure S9

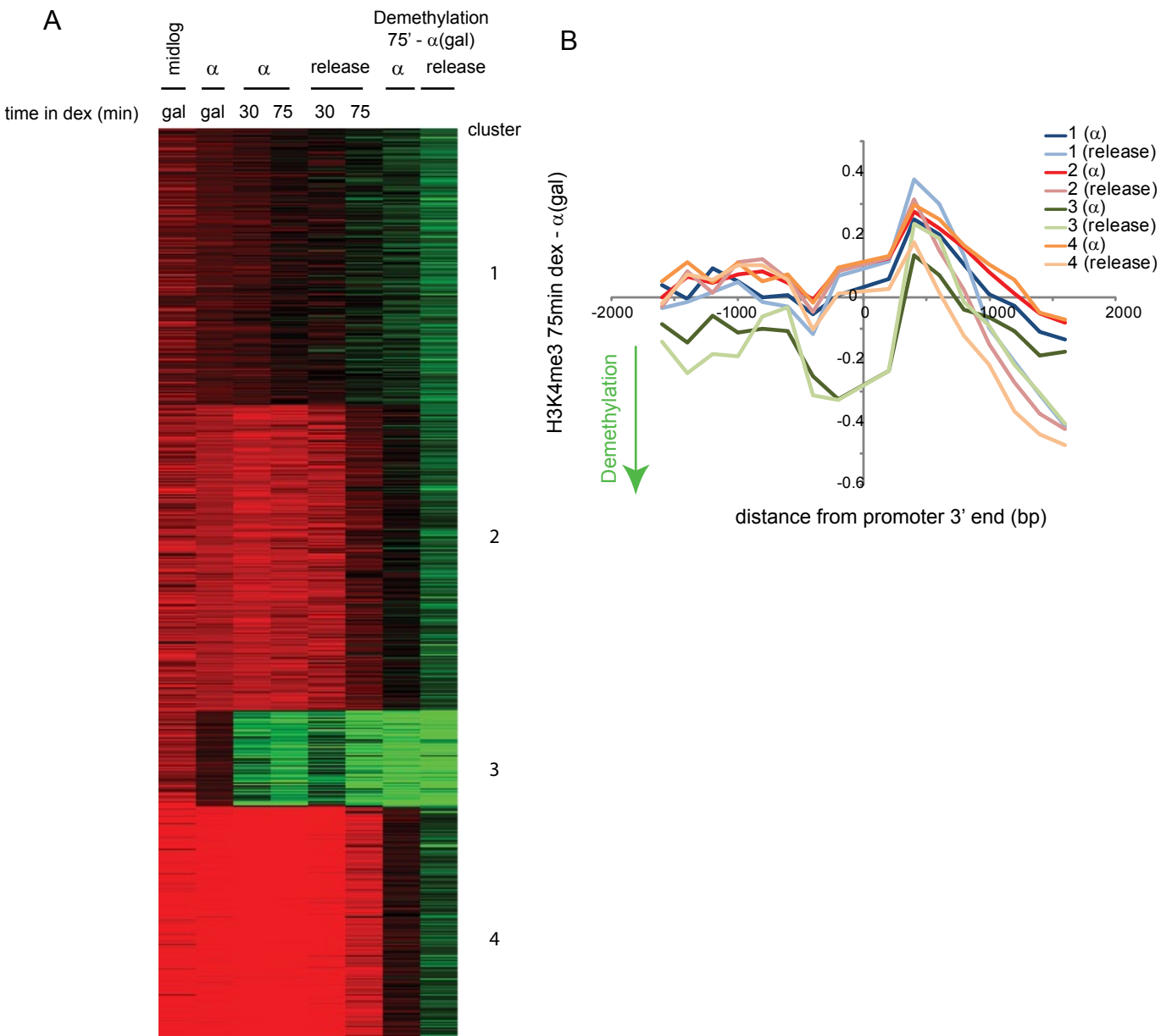

Supplement: Figure S9 — Efficient S phase-independent H3K4me3 erasure occurs preferentially at 5′ ends of coding regions. (A) Whole-genome microarray data for wild-type cells grown as indicated in Figure 6D. Probes exhibiting above-average H3K4me3 levels during midlog growth, but which did not gain H3K4me3 during alpha factor arrest, were subjected to k-means clustering with k = 4. Cluster 3 probes indicate probes that lose H3K4me3 upon addition of dextrose, independent of alpha factor release (compare rightmost two columns). Conversely, Cluster 1, 2, and 4 exhibit greater demethylation upon alpha factor release. (B) 3′ coding regions exhibit release-dependent demethylation. Probes from Clusters 1–4 were ordered according to distance from transcription start site (x axis), and average H3K4me3 loss 75 minutes after dextrose addition is shown for continued arrest (α) or concomitant release. Note that Cluster 3 nucleosomes (green) exhibit release-independent 5′ demethylation, whereas all clusters showed release-dependent 3′ demethylation. (0.33 MB PDF) [file pgen.1000837.s009.pdf]

Figure S10

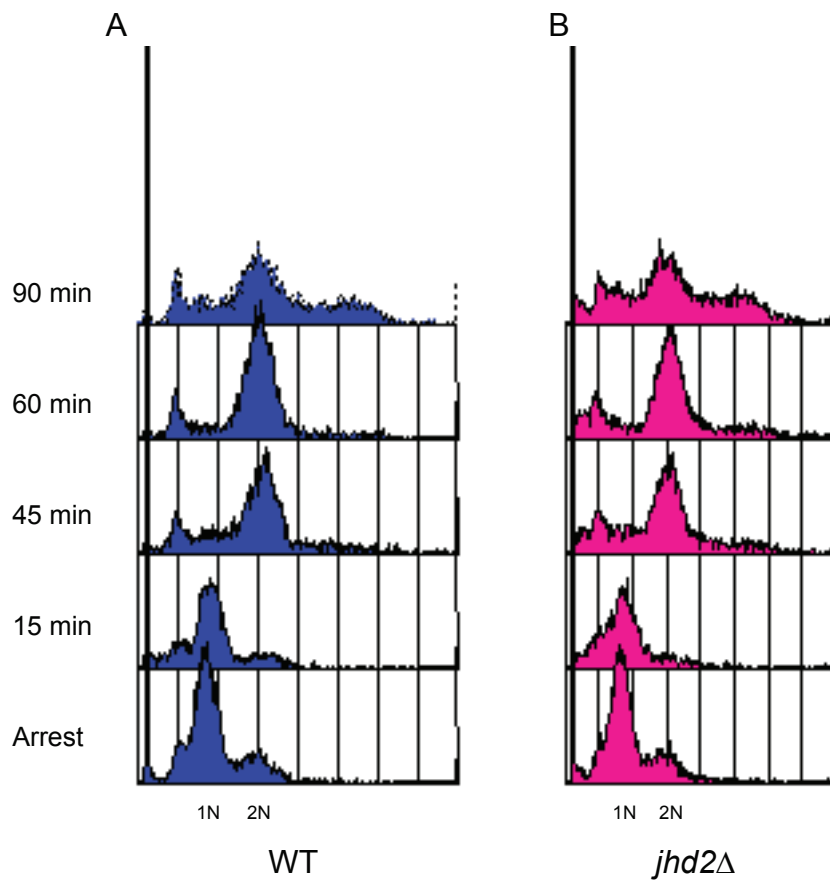

Supplement: Figure S10 — Jhd2 does not affect cell cycle synchrony. FACS analysis of wild-type (A) and jhd2Δ (B) yeast during alpha factor arrest, and at varying times after release. (0.26 MB PDF) [file pgen.1000837.s010.pdf]

Figure S11

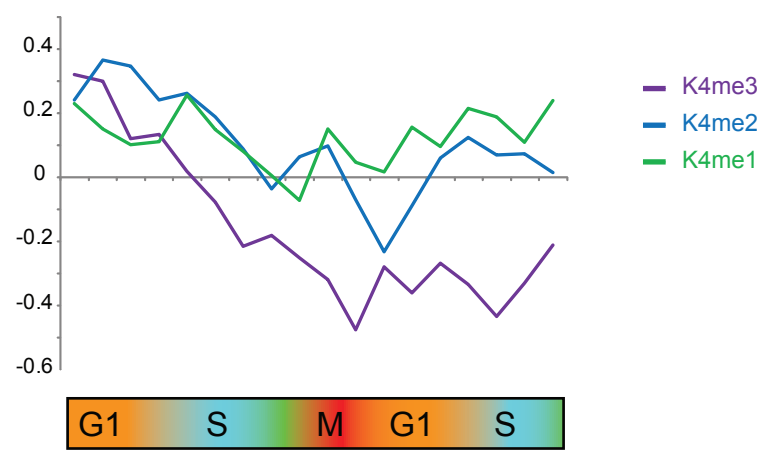

Supplement: Figure S11 — Progressive demethylation at H3K4. Averaged data for H3K4me3, me2, and me1 are shown for Cluster 6 nucleosomes during a replicate of the CCTS time course. (0.22 MB PDF) [file pgen.1000837.s011.pdf]

Figure S12

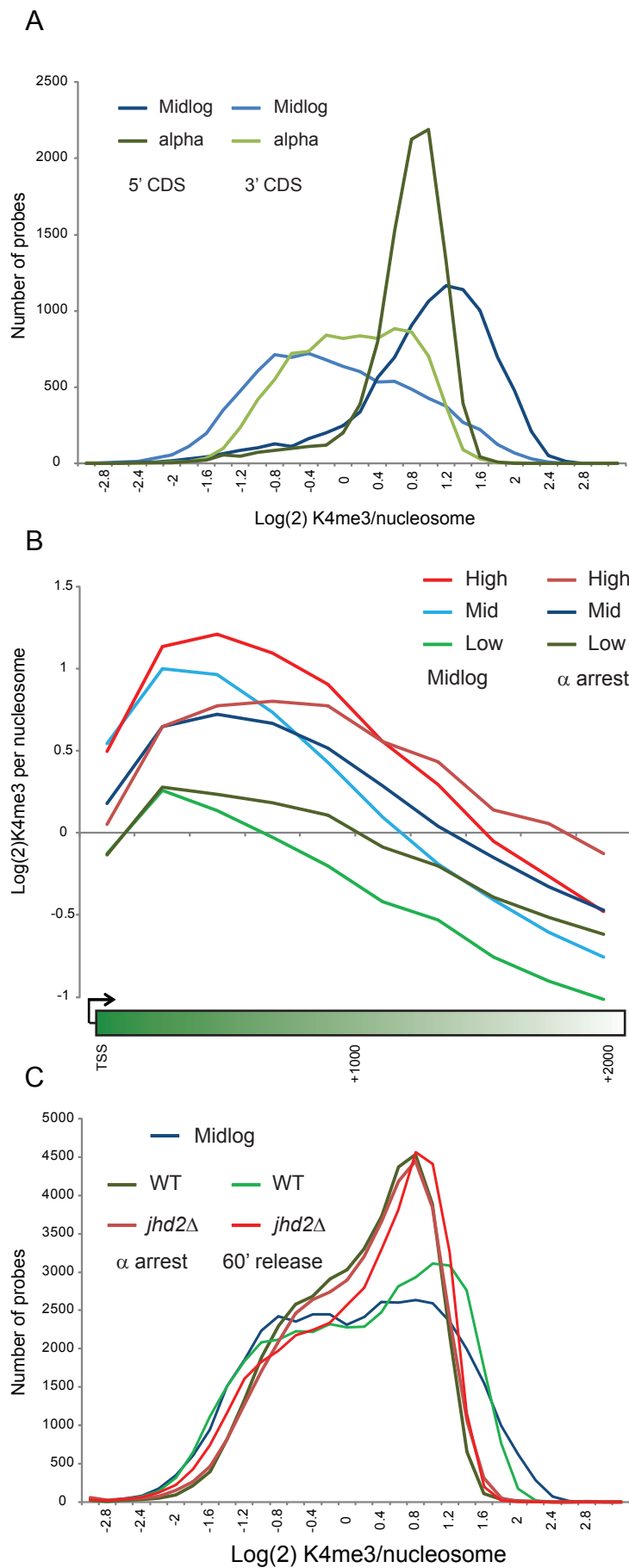

Supplement: Figure S12 — Global changes in H3K4me3 during alpha factor arrest. (A) Coding region H3K4me3 flattens during alpha factor arrest. Distributions of H3K4me3/nucleosome were calculated for 5′ and 3′ probes during midlog and alpha factor arrest (indicated), showing that 3′ probes, which are normally hypomethylated become more methylated during arrest. (B) Alpha factor arrest results in H3K4me3 extension into coding regions. Averaged data for high, middle, and low expression level genes are shown with probes ordered according to distance into coding region. Note that during alpha factor arrest the normally 5′-biased H3K4me3 pattern extends further into coding regions, particularly at poorly-expressed genes. (C) Jhd2 is required to counteract alpha factor-dependent H3K4me3 flattening. As in (A), except all genomic probes are included in histograms. Note that both in wild-type and jhd2Δ yeast H3K4me3 distributions become more unimodal during alpha factor arrest, and this is reversed upon release in a Jhd2-dependent manner. (0.31 MB PDF) [file pgen.1000837.s012.pdf]
